# TLR2 and TLR4 in the Brain Injury Caused by Cerebral Ischemia and Reperfusion

**DOI:** 10.1155/2013/124614

**Published:** 2013-06-23

**Authors:** Ying Wang, Pengfei Ge, Yuhong Zhu

**Affiliations:** ^1^Department of Neurology, Second Affiliated Hospital of Kunming Medical University, Kunming 650031, China; ^2^Department of Neurosurgery, First Hospital of Jilin University, Changchun 130021, China

## Abstract

Brain injury caused by cerebral ischemia/reperfusion is a complicated pathophysiological course, in which inflammation is thought to play an important role. Toll-like receptors are a type of transmembrane proteins, which can recognize either exogenous pathogen-associated molecular patterns or endogenous stress or damage-associated molecular patterns in the innate immune system and initiate inflammatory responses. Among Toll-like receptors, TLR2 and TLR4 are found to be more important than others in the pathological progression of cerebral injury due to ischemia and reperfusion. This review will focus on the biological characteristics and functions of TLR2 and TLR4 and their downstream signal pathways.

## 1. Introduction

In 1980, a German biologist Nusslein-Volhard found a mutant gene in the embryos of Drosophila melanogaster, which was associated with regulating Drosophila dorsal-ventral polarity and was named “Toll gene” [1]. In 1984, Steward reported a “Toll protein” in Drosophila melanogaster, which was not only responsible for promoting dorsal-ventral differentiation, but could also recognize the invaded microbes and initiate antibacterial process [2]. In 1997, Janeway and Medzhitov reported firstly that a receptor, known currently as TLR4, was located on the human cell surface and was functionally similar to Drosophila Toll protein [3]. Thereafter, Medzhitov found some similar receptors in other animals or plants and named them Toll-like receptors (TLRs). By now, 10 functional TLRs in humans and 12 in rodents have been identified, respectively [4], of which TLR1-TLR9 were shared by both humans and rodents. Although it was initially thought that TLRs were associated with the development of mammals, Jules Hoffmann and Bruce Beutler found that TLRs are key sensors to infectious microorganisms in the mammalian innate immune response. Moreover, TLRs modulate the expression of inflammatory mediators when they were activated by host-derived molecules [5]. 

The mechanism underlying brain injury caused by cerebral ischemia and reperfusion remains elusive, but inflammatory response has been found to be one of the main factors leading to brain damage [6, 7]. Accumulating evidences show that TLRs (Toll-like receptors) are activated by endogenous proteins released from damaged brain and played crucial role in mediating the cerebral injury following ischemia and reperfusion [8]. In particular, TLR2 and TLR4 were found to be more important than other TLRs in the pathologic progression of cerebral ischemia and reperfusion [7, 9]. Therefore, TLRs have become a potential target in searching new strategy for the treatment of ischemic cerebrovascular disease. This review will focus on the biological characteristics and functions of TLR2 and TLR4 and their downstream signaling pathway.

## 2. Structure of Toll-Like Receptors

Toll-like receptors belong to the type I transmembrane glycoprotein receptor family. They are composed of extracellular N-terminal ligand-recognition domain, transmembrane domain, and intracellular C-terminal signaling domain [10]. The extracellular domain contains 16–28 extracellular leucine-rich repeat (LRR) modules and is mainly responsible for binding to exogenous pathogen-associated molecular patterns (PAMPs) [11] and endogenous stress or damage-associated molecular patterns (DAMPs) [12]. The intracellular domain of TLRs is also known as Toll/IL-1 receptor (TIR) domain, because highly conserved sequences within it shared homologous to those of human cytoplasmic interleukin-1 receptor (IL-1R). Extracellular signals enter into cell and initiate the “signaling cascades” when the TIR domain was attached by intracellular specific adaptors [13], which include Myeloid differentiation factor 88 (MyD88), TIR domain-containing adaptor protein (TIRAP)/MyD88 adaptor-like (Mal), TIR domain-containing adaptor-inducing IFN-*β* (TRIF), TRIF-related adaptor molecule (TRAM), and Sterile *α* and armadillo motif-containing protein (SARM).

## 3. TLR2/TLR4 and Their Ligands in Cerebral Ischemic Reperfusion Injury

Toll-like receptors are found to present on various innate immune cells, such as polymorphonuclear neutrophils, monocyte/macrophage, dendritic cells, and NK cells, in which they trigger an immediate response against pathogens [14–16]. Within brain, they are mainly located on glial cells including microglia, astrocytes, and Oligodendrocytes. Microglia and astrocytes express a wide repertoire of TLRs [17], and they both could produce proinflammatory cytokines when TLRs are attached with their corresponding ligands [18–20]. By contrast, Oligodendrocytes can express a little repertoire of TLRs such as TLR2 and TLR3, which is mainly involved in central nervous system repair [21]. During recent years, the reports involving the expression of TLRs in neurons have sharply increased as well. 

Although the central nervous system is a sterile circumstance and no pathogens such as germs or viruses exist under normal or ischemic condition, it is found that cerebral ischemia causes elevation in the expression of TLR2, TLR4, and TLR9 in neurons [22, 23]. By contrast, accumulating experimental evidences suggested that TLR2 and TLR4 play crucial roles in modulating inflammatory response caused by cerebral ischemia and reperfusion via linking to their endogenous ligands, respectively [24–26]. These endogenous ligands include heat-shock proteins (HSPs), high mobility group box 1 (HMGB1), hyaluronic acid, fibronectin [27, 28], and so on. 

Heat shock proteins (HSPs) are normally in small quantity and function as chaperone to maintain effectively protein configuration. Heat shock protein family includes several members, such as HSP60, HSP70, and gp90. However, under the stress or damage conditions such as ischemia or hypoxia, their expression could be upregulated and they leak into the extracellular compartment to induce immune response and inflammatory response as endogenous ligands. During the course of cerebral ischemic/reperfusion injury, the binding of HSPs to TLR2 and TLR4 could initiate the signal pathway related to proinflammatory cytokines production [29]. Of all HSPs members, HSP60 and HSP70 are the most important endogenous ligands to TLR2 and TLR4. The binding of HSP60 with TLR2 and TLR4 leads to the recruitment of MyD88, activation of transcription factor, and increased expression of inflammatory cytokines TNF-*α* [30]. Similarly, the binding of HSP70 with TLR2 and TLR4 activates the MyD88-IRAK-NF-*κ*B signal pathways and promotes the transcription and expression of TNF-*α*, IL-1*β*, and IL-6 [31]. Brea et al. demonstrated in their vitro model study that blocking either HSP60 or TLR2 and TLR4 suppressed the strong inflammatory response in the cultures cells (monocytes and human umbilical vein endothelial cells) treated with serum from ischemic stroke patients [32]. This indicated that HSP60 might be a new therapeutic target for ischemic stroke.

High mobility group box 1 (HMGB1) belongs to the nuclear nonhistone DNA-binding protein family, which is responsible for maintaining the configuration of DNA in the nucleus and regulating gene transcription under normal conditions. HMGB1 participates in the inflammatory response under stress or damaging conditions when they leak into the extracellular compartment [33]. Peltz et al. found that plasma HMGB1 was markedly elevated within 2 to 6 hours in the acute damage event [34]. By using immunoprecipitation, researchers showed that HMGB1 can indeed be involved in the inflammatory responses through interacting with TLR2 and TLR4 [35]. The combination of HMGB1 with TLR2 or TLR4 activated its downstream MyD88-TIRAP-IRAK signal pathway to induce the release of cytokines such as TNF-*α*, iNOS, and ICAM-1. Moreover, the experimental data presented by Qiu et al. showed that the increased expression of MMP-9 in neurons and astrocytes following cerebral ischemia was triggered by HMGB1 predominantly via TLR4 pathway. Similarly, it was reported that the level of TLR4 increased in the primary cultured neurons and astrocytes treated with HMGB1. In the TLR2 overexpressional cells, HMGB1 can effectively induce the release of IL-8 and TNF-*α*. Thus, these studies showed that HMGB1 induce inflammatory response via TLR2 or TLR4 pathway. However, evidences from in vitro and in vivo studies showed that downregulating the expression of HMGB1 in neural cells was associated with the protection of short hairpin RNA (sh RNA) on ischemic neurodegeneration. It was also found that inhibition of HMGB1 expression reduced neuronal death, attenuated the activation of immune glia, and suppressed the induction of proinflammatory mediators such as iNOS, COX-2, IL-1*β*, and TNF-*α* in postischemic brain [36]. Particularly, in the case of transient middle cerebral artery occlusion (MCAO), the infarct volume was found to be markedly smaller in TLR4 mutant mice than that in the wild-type mice [37]. Anti-TLR2 antibodies diminished the expression of IL-8 and TNF-*α* via inhibiting the function of HMGB1 [38]. Therefore, as we discussed earlier, HMGB1 is an important ligand to bind with TLR2 and TLR4 and regulates the production of inflammatory factors. 

Fibrin/fibrinogen is an acute reactive protein synthesized and secreted by hepatocytes and associated with coagulation process under normal condition. However, under stress or hypercoagulating state, fibrin/fibrinogen content will increase significantly and become a crucial factor in regulating the formation of thrombosis. Cerebral ischemic/reperfusion could induce accumulation of vascular fibrin/fibrinogen, which causes either the formation of microthrombosis resulting in microcirculation disorders, or upregulation in the expression of TLR4 and IRAK1 (interleukin-1 receptor activated kinases 1) on the cerebral endothelial cells. Meanwhile, acute accumulation of fibrin/fibrinogen has been shown to induce proinflammatory responses as well as disruption in the blood-brain barrier via TLR signaling pathway during the course of cerebral ischemic insult [39, 40]. Zhang et al. demonstrated that the vessels with immunoreaction to fibrin/fibrinogen showed positive immunoresponses as well to TLR2, TLR4, and IRAK1, which suggested that the deposition of fibrin/fibrinogen on cerebral vessels induced by stroke could trigger the expression of TLRs. Combination therapy with VELCADE (a potent proteasome inhibitor) and tissue plasminogen activator (tPA) significantly reduced lesion volume and improved neurological score in MCAO rats. The authors thought that the therapy with VELCADE and tPA abolished the inactivation of fibrin/fibrinogen on NF-*κ*B by directly inhibiting stroke-activated TLR2, TLR4, and IRAK1 and suppressing inflammatory respond [41].

Additionally, low molecular weight hyaluronan is also an endogenous ligand to TLR2 and TLR4. It activates the innate immune response via MyD88, IRAK, TNF-receptor association factor 6 (TRAF-6), and NF-*κ*B pathway.

## 4. TLR2 and TLR4 Signaling

TLRs signal activates the transcription factors and generates cytokines and chemokines via intracellular pathways. TLR2 and TLR4 combined with their respective ligands to form dimeric complexes and change their configuration and then recruit five specific adaptors within cells including MyD88, TIRAP/Mal, TRIF, TRAM, and SARM.

**Figure 1:**
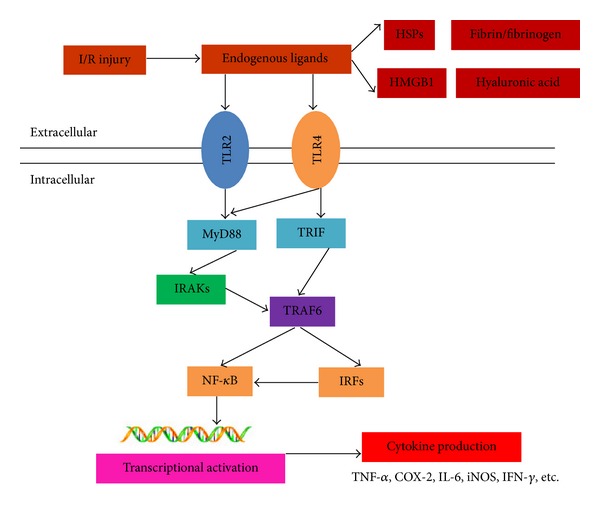
Brain injury caused by cerebral ischemia/reperfusion is a complicated pathophysiological course, in which TLR2 and TLR4 are thought to play an important role. TLR2 and TLR4 play crucial roles in modulating inflammatory response caused by cerebral ischemia and reperfusion via linking to their endogenous ligands and then recruit specific adaptors within cells. TLRs signal activates the transcription factors and generates cytokines and chemokines via intracellular pathways.

TLR2 and TLR4 bind to these specific adaptors via their unique TIR domains. Depending on different adaptor proteins, the extracellular signals are transmitted to the downstream modules mainly via MyD88-dependent and MyD88-independent pathways (TIRAP/Mal, TRIF, TRAM, and SARM) (see Figure 1).

In MyD88-dependent pathway, MyD88 death domain interacted with interleukin-1 receptor-associated kinases (IRAKs) to activate TRAF-6. Following being phosphorylated by TRAF-6, the inhibitory *κ* kinases *α*/*β* (I*κ*K *α*/*β*) activated transcription factor NF-*κ*B and interferon promoter-binding protein (IRFs). Then, NF-*κ*B and IRFs translocate into nucleus and induce the generation of a variety of cytokines, such as TNF-*α*, IL-1*β*, and IFN-*β*. TLR2 mainly transmits signals via MyD88-dependent pathway. In MyD88-independent pathway, TRIF interacts with TRIF-related adaptor molecule (TRAM), activates TRAF3, and phosphorylates I*κ*K*ε*. Finally, phosphorylated I*κ*K*ε* activates IRF3 and IRF7 and generates IFN-*β*. Besides MyD88 dependent and independent signal pathways, TLR4 mediates cell proliferation, transformation, and apoptosis via mitogen-activated protein kinases (MAPKs) signaling pathway [42].

## 5. TLR2 in Cerebral Ischemic/Reperfusion Injury

TLR2 is an important member in brain innate immune response system and mainly expressed on microglia, astrocyte, neuron, and endothelial cell. These glial cells could secrete the inflammatory factors, as well as generate proinflammatory and proapoptotic mediators related to TLR2 gene to exacerbate brain damage. To examine whether the TLR2 protein is of functional relevance to cerebral ischemia, brain damage was compared at reperfusion 2 days following 1 h MCAO in TLR2-deficient mice and wild-type mice. The result showed that the infarct volume in the TLR2-deficient mice was significantly smaller than that in the wild type mice [43]. Moreover, Lv et al. reported that sequential expressions of TLR2, IL-23, and IL-17 were observed either in microglia after cerebral ischemia/reperfusion or in cells cultured under the condition of oxygen-glucose deprivation reperfusion (OGDR) [44]. This study showed that microglia activated by ischemia/reperfusion or OGDR aggravated neuronal damage via secretion of toxic cytokines IL-23 and IL-17. By contrast, suppression of TLR2-IL-23-IL-17 axis in microglia leaded to reduction in neuronal apoptosis caused by OGDR or ischemia/reperfusion. These studies indicated that TLR2 plays a critical role in cerebral ischemic/reperfusion injury and initiates the inflammatory cascade to exacerbating brain damage. In the brain, the scavenger receptor CD36 is required for the inflammatory response triggered by TLR2 signaling. In the CD36 null mice, transient MCAO did not induce the expression of inflammatory genes thought to be upregulated by TLR2 and to aggravate brain damage. Thus, this indicated that TLR2-CD36 complex is a sensor of ischemia-induced prodeath signals and is critical for the inflammatory response [45]. Moreover, the same conclusion has achieved in accumulating experiments that TLR2 is a critical inflammatory trigger in cerebral ischemic/reperfusion injury [46, 47].

## 6. TLR4 in Cerebral Ischemic/Reperfusion Injury

TLR4 is a crucial receptor in the innate immune system and noninfectious immune responses. To clarify whether each of Toll-like receptors participate in the pathological course of cerebral ischemia/reperfusion injury, the size of brain infarction was compared by using TLR3, TLR4, TLR9 knock-out mice, and wild-type mice. The result showed that only the TLR4 knock-our mice has significantly smaller infarct volume at reperfusion 24 h after 2 h ischemia, when compared with wild-type mice [48]. This study suggested that TLR4 might play more important role than other Toll-like receptors during the course of brain damage caused by ischemia/reperfusion. The level of TLR4 mRNA increased in neurons after 1 h of cerebral ischemia, which was accompanied by the high level of multiple inflammatory cytokines [47]. Moreover, it was reported that LPS can serve as a potent preconditioning stimulus and provide protection against ischemic brain injury via modulating TRL4 [49]. Similarly, other experiments revealed that TLR4 could regulate the expression of cytokines, such as TNF-*α*, COX-2, IL-6, iNOS, and IFN-*γ* [50–53].

## 7. TLR2/TLR4 and Cytokines

Cerebral ischemic/reperfusion injury is accompanied with the inflammatory responses, which induce production of chemokines, adhesion molecules, and a large number of proinflammatory factors. IL-1*β* is an immune-derived cytokines and could be detected in brain at 30 min reperfusion after cerebral ischemia, which directly induces neuronal apoptosis and enhances the expression of chemokines within microglia and astrocyte. IL-1*β* possesses autocrine-like function and promotes the secretion of itself under ischemic stimuli. It is also considered to be a neurotoxic mediator, because infarct volume would decrease when its function is lost. In most cases, endogenous ligands binding to TLRs activate monocytes/macrophages to secrete IL-1*β*. Abulafia et al. reported that the level of IL-1*β*mRNA decreased in the TLR2 and TLR4 deficient mice which suffered from thromboembolic stroke [54].

TNF-*α* is another inflammatory cytokine participating in the pathological process of cerebral ischemia/reperfusion. Under normal circumstance, TNF-*α* is correlated with immune responses and could repair the cells of nervous system. It was found that the expression of TNF-*α* gradually increases in cerebral ischemia at 1 h reperfusion following cerebral ischemia. TNF-*α* promotes release of excitatory amino acid and production of oxygen free radicals. During the inflammatory procedure, TNF-*α* induces microglia and astrocyte to express cytotoxic iNOS. Meanwhile, the infiltration of leukocytes into brain parenchyma to aggravate ischemic injuries is also due to the assistance of TNF-*α*. Tu et al. used rat model of permanent MCAO to demonstrate that inhibition of TLR2 and TLR4 signaling pathway by baicalin reduced the volume of infarct brain and the serum level of TNF-*α*, IL-1*β*, and iNOS via inhibiting using [55].

IL-6 possesses multiple biological functions and has been mainly secreted by astrocyte and microglia during the course of cerebral ischemia/reperfusion. Moreover, a variety of cytokines can induce the production of IL-6 as brain tissue suffered from the damage stimulus. IL-6 can be detected in the brain tissue, cerebrospinal fluid, and serum at reperfusion 2 h after cerebral ischemia. However, some studies have shown that IL-6 could stimulate astrocyte to generate neurotrophic factors and nervous growth factors, which assist the repair of damaged nervous cells via JAK or STAT pathways but also participate in the inflammatory injuries as well [56]. Cao et al. demonstrated, by using mice model of MCAO, that the neurological impairments and the level of IL-6 were both improved more obviously in TLR4 deficient mice than in wild-type mice [51]. 

Chemokine is a family of small cytokines and can enhance inflammatory response. Meanwhile, chemokine could act as a chemoattractant to guide the migration of cells and promote leukocytes infiltrating into brain tissue during the process of cerebral ischemia and reperfusion. It was found that the proliferation capacity of resident microglia and the level of monocyte chemotactic protein-1 (MCP-1) were both reduced in the TLR2 deficient mice treated with transient MCAO, when compared with that in the wild-type mice [57]. Adhesion molecules are located on cell surface to assist cells sticking to each other and their surroundings. For an instance, adhesion molecule ICAM could induce leukocyte rolling and sticking to the vascular endothelial cells, which lead to the penetration of leukocyte into extravascular matrix to release cytotoxic protease and aggravation of brain injuries caused by cerebral ischemia/reperfusion. In vitro, treatment with rhHMGB1 made the expression of TNF-*α* and ICAM-1 up-regulated in the primary cultured neurons and endothelial cells that expressed TLR2 and TLR4 [58]. Again, TLR2 and TLR4 binding to HMGB-1 indeed promotes inflammatory responses.

## 8. Inference

There is a definite conclusion on the basis of current findings that TLR2 and TLR4 exert key influences on the pathological process of cerebral ischemia/reperfusion. The common characteristics are that (1) the expressions of TLR2 and TLR4 increase at the beginning of reperfusion following cerebral ischemia and last a long time; (2) the methods inhibiting the expression of TLR2 and TLR4 in brain tissue have significantly suppressed neurological deficits and alleviated brain damage caused by cerebral ischemia and reperfusion; (3) the expressional level of TLR2 and TLR4 influenced the production of multiple cytokines which participate in inflammatory signal pathway and decided the outcome of cerebral ischemia and reperfusion. Clinical study showed that the level of TLR2 and TLR4 in neutrophils at 72 h and 7 days following ischemia and reperfusion was an independent indicator associated with the volume of brain infarction and prognosis of stroke patients [59]. Moreover, it was found that the level of TLR4 mRNA elevated significantly within peripheral blood in patients with transient ischemic attack (TIA) or stroke when compared with that in the patient with asymptomatic internal carotid artery stenosis [60]. Therefore, inhibition of TLR2 and TLR4 has become a strategy to prevent or treat ischemic cerebrovascular diseases.

## 9. Prospective

Some compounds or chemicals such as baicalin, luteolin, and picroside II have been selected to inhibit TLR2 and TLR4. Neurological deficit, infarct volume, the expression of TLR2, TLR4, and a variety of inflammatory cytokines in the damaged brain tissue were examined at different reperfusion time points by using rat MCAO model. The results showed that most of previously mentioned compounds could alleviate brain damage caused by cerebral ischemia/reperfusion via inhibiting the expression of TLR2, TLR4, and inflammatory cytokines NF-*κ*B, iNOS, COX-2, IL-6, and TNF-*α* [55, 61, 62].

By contrast, some ligands of TLR4 or TLR2 could be used as preconditioning inducer, which would enhance cerebral resistance to severe ischemia and reperfusion. The mice pretreated at 72 h prior to cerebral ischemia and reperfusion with subcutaneous injection of low dose of LPS, a specific ligand of TLR4, showed decreased brain infarction. The possible mechanisms are considered to be via suppressing TLR4-related inflammatory signals such as MyD88-TRIF-IRF3-NF-*κ*B pathway [63], or inducing production of neuroprotective cytokines TGF-*β* and IL-10. Similarly, preconditioning with TLR2 specific ligand, Pam_3_CSK_4_, could significantly reduce neuronal apoptosis and brain damage caused by cerebral ischemia/reperfusion via activation of TLR2-PI3K-Akt pathway [64].

Experimental and clinical studies have demonstrated that physical exercise benefits neurological recovery or reduction of neurological dysfunction caused by cerebral ischemia and reperfusion. Mild treadmill training at three weeks prior to brain ischemia, or at day 5 reperfusion following ischemia, contributed to the recovery of neurological dysfunction via inhibiting the mRNA level and protein concentration of TLR2 and TLR4 and their downstream molecules (such as MyD88 and NF-*κ*B) in rat brain tissue [65, 66].

Acupuncture is an important procedure in traditional Chinese medicine and has been used as a complementary and alternative treatment for neurological recovery. Electric stimulation to some acupoints (such as Quchi and Zusanli) has been demonstrated to be neuroprotective and anti-inflammatory. Further study showed that the effects of electroacupuncture on improving significantly the neurological deficits, reducing infarct brain tissue, and alleviating inflammatory responses were via inhibiting TLR4 signaling pathway [67].

Moreover, other means have also been exerted to suppress overexpression of TLR2 and TLR4 during cerebral ischemia/reperfusion. As mentioned earlier, CD14 is an important molecule to assist the formation of TLR4 homodimer. Canavanine, an iNOS selective inhibitor, not only inhibited the elevation of CD14 and TNF-*α* in microglia exposed to hypoxia circumstance, but also suppressed TLR4 to form homodimer and blocked its downstream signaling pathways [68].

## 10. Conclusions

Brain injury caused by cerebral ischemia/reperfusion is a complicated pathological course, in which inflammatory response plays a crucial role. Moreover, accumulating evidences showed that TLR2 and TLR4 are the most important Toll-like receptors during this process. A variety of means have been used to demonstrated the effectiveness of inhibiting the expression of TLR2 and TLR4 and their downstream signaling pathways on protecting brain injury caused by cerebral ischemia and reperfusion. However, further studies are also needed to screen out a medicine targeting to block TLR2 and TLR4 and could be used in future clinical practice.
